# Vulnerabilidades e estereótipos masculinos nas representações sociais das causas do adoecimento por câncer de próstata

**DOI:** 10.1590/0102-311XPT175123

**Published:** 2024-10-11

**Authors:** Widson Davi Vaz de Matos, Iaci Proença Palmeira, Márcia de Assunção Ferreira, Mayara Del Aguilal Pacheco

**Affiliations:** 1 Universidade do Estado do Pará, Belém, Brasil.; 2 Universidade Federal do Rio de Janeiro, Rio de Janeiro, Brasil.

**Keywords:** Neoplasias de Próstata, Saúde do Homem, Psicologia Social, Prostatic Neoplasms, Men’s Health, Social Psychology, Neoplasias de la Próstata, Salud del Hombre, Psicología Social

## Abstract

No universo consensual de pensamento, o câncer é associado à doença incurável e incapacitante, o que acarreta prejuízos que extrapolam o âmbito biológico e atinge dimensões psicoculturais e sociais de quem convive com a doença. Sob a ótica cultural, os homens constroem narrativas sobre o câncer de próstata com base nas suas experiências e contextos sociais, expressando elementos morais, éticos e sociopolíticos atribuídos à causa do adoecimento por esse tipo de câncer. O objetivo do estudo foi compreender as causas do adoecimento de câncer de próstata nas representações de homens acometidos desse tipo de câncer e suas repercussões no autocuidado. Realizou-se estudo descritivo, qualitativo, fundamentado na Teoria das Representações Sociais. Participaram 31 homens com diagnóstico de câncer de próstata matriculados em uma Unidade de Alta Complexidade em Oncologia. Os dados foram produzidos de abril a junho de 2022 por entrevista em profundidade, individual e semiestruturada. O *corpus* foi submetido ao software ALCESTE. A ingestão de bebida alcoólica, o tabagismo, a promiscuidade sexual e a ausência de autocuidado foram os principais comportamentos entendidos como causas do câncer, o que gera autorresponsabilização e culpa pelo adoecimento. Representações sociais das causas, traduzidas em comportamentos não alinhados ao que a moral social dita como certos, repercutem na noção moralizadora do câncer como um castigo, em que a doença expressa o caráter do paciente, ancorando-se no discurso religioso judaico-cristão, o que diminui a carga sociopolítica das vulnerabilidades masculinas e reforça estereótipos da sociedade patriarcal.

## Abstract (en)

In society, cancer is commonly associated with an incurable and disabling disease that causes damage beyond the biological scope, impacting the psychological and sociocultural dimensions of cancer patients. From a cultural point of view, men construct narratives about prostate cancer based on their experiences and social contexts, expressing moral, ethical, and sociopolitical elements attributed to the cause of this type of cancer. We sought to understand the causes of prostate cancer as represented by men living with this type of cancer and its repercussions on self-care. A descriptive, qualitative study based on the Theory of Social Representations was conducted with 31 men diagnosed with prostate cancer treated at a High Complexity Oncology Unit. Data were collected from April to June 2022 by in-depth, individual, and semi-structured interviews. The corpus was inserted into the ALCESTE software. Alcohol intake, smoking, sexual promiscuity, and not taking care of oneself were the main behaviors understood as causes of cancer, which generates self-responsibility and guilt for the illness. Social representations of these causes, translated into behaviors not aligned with what social morality dictates as right, have repercussions on the moralizing notion of cancer as punishment, in which the disease expresses the patient’s character, anchored in the Judeo-Christian religious discourse, which reduces the sociopolitical burden of male vulnerabilities and reinforces stereotypes from patriarchal society.

## Resumen

En el universo de pensamiento consensuado, el cáncer se asocia a una enfermedad incurable e incapacitante, que provoca pérdidas que van más allá del ámbito biológico y afectan las dimensiones psicoculturales y sociales de quienes viven con la enfermedad. Desde una perspectiva cultural, los hombres construyen narrativas sobre el cáncer de próstata con base en sus experiencias y contextos sociales, expresando elementos morales, éticos y sociopolíticos atribuidos a la enfermedad por este tipo de cáncer. El objetivo fue comprender las causas de la enfermedad de cáncer de próstata en las representaciones de los hombres afectados por ese tipo de cáncer y sus repercusiones en el autocuidado. Estudio descriptivo, cualitativo, basado en la Teoría de las Representaciones Sociales. Participaron 31 hombres diagnosticados con cáncer de próstata inscritos en una Unidad de Oncología de Alta Complejidad. Los datos se produjeron de abril a junio del 2022 mediante entrevistas en profundidad, individuales y semiestructuradas. El *corpus* fue sometido al software ALCESTE. Consumir alcohol, fumar, la promiscuidad sexual y no cuidarse fueron las principales conductas entendidas como causantes del cáncer, lo que genera autorresponsabilidad y culpa por la enfermedad. Las representaciones sociales de las causas, traducidas en comportamientos no alineados con lo que la moral social dicta como correctos, repercuten en la noción moralizante del cáncer como castigo, en que la enfermedad expresa el carácter del paciente, anclándose en el discurso religioso judeocristiano, lo que disminuye la carga sociopolítica de las vulnerabilidades masculinas y refuerza los estereotipos de la sociedad patriarcal.

## Introdução

As neoplasias se configuram como problema de saúde pública no Brasil, motivado pela magnitude epidemiológica e pelo impacto econômico e social, por ser uma doença crônico-degenerativa ^1^. No Brasil, estima-se que, para o triênio 2023-2025, ocorrerão 71.730 casos novos de câncer de próstata ^2^, ocupando a segunda posição entre os tipos mais frequentes de cânceres e o mais incidente nas regiões do país, com um risco estimado de 28,40 casos a cada 100 mil homens na Região Norte. No Pará, estima-se que ocorram 1.050 casos novos de câncer de próstata, sendo 130 casos na capital do estado, Belém ^2^.

As crenças sociais preconcebidas associadas ao câncer, concatenadas aos impactos físicos gerados pela doença, como alteração da imagem corporal, mal-estar, possível submissão a tratamentos que levam a alopecia, náuseas, fadiga, acarretam prejuízos que extrapolam o âmbito meramente biológico e atingem dimensões psicossociais e culturais, afetando a vida e o cotidiano das pessoas acometidas ^3^. Nesse contexto, o câncer de próstata comporta-se como um fenômeno e objeto capaz de gerar processos representacionais que auxiliam os homens na compreensão da nova realidade e orientação de condutas diante do adoecimento.

Devido às crenças, cultura e trabalho, os homens buscam menos os serviços de saúde, culminando, quase sempre, em diagnósticos com estágios avançados da doença e reduzidas possibilidades de tratamento, cura e reabilitação ^4^. Destaca-se que os modos pelos quais os homens concebem e vivenciam suas masculinidades podem interferir diretamente nos processos saúde-doença e práticas de cuidado ^4^ ^,^ ^5^. Ressalta-se o emprego da categoria “masculinidade” no plural, por considerar as múltiplas possibilidades de sua expressão social ^6^.

Em 2009 o Ministério da Saúde elaborou a Política Nacional de Atenção Integral à Saúde do Homem (PNAISH), que, entre outras diretrizes, preconiza a necessidade de os profissionais de saúde observarem a população masculina com lentes contemporâneas de pensamentos, contribuindo para o fortalecimento da adesão aos processos de acolhimento e abordagem desse público no processo de saúde ^7^.

O homem deve ser visto como um ser singular com necessidades específicas de cuidados. Considerando a saúde do homem e os altos coeficientes de morbimortalidade masculina por câncer de próstata, torna-se importante que a equipe de saúde se interesse pelos saberes de homens com câncer de próstata, para acessar o que pensam sobre a doença, o adoecimento, o autocuidado, quais sentimentos permeiam a vivência do adoecimento, com vistas às reflexões sobre a prevenção e o tratamento desta enfermidade. Acessar tais saberes viabiliza que emerjam subsídios aos cuidados de saúde para além da abordagem biomédica, com potencial abrangência de aspectos biopsicossociais de homens com câncer de próstata, justificando assim a realização deste estudo.

Pesquisa realizada com equipe de saúde de uma localidade da Região Nordeste do Brasil indicou que o medo, a vergonha e o preconceito em relação ao exame de próstata incidem sobre a prevenção de câncer de próstata ^8^, o que reforça a relevância desse tema. Portanto, acessar os saberes de homens com câncer de próstata sobre essa patologia, requer um olhar que contextualize esse homem social e culturalmente, para se compreender como ele é investido de representações sociais e como estas repercutem nas ações de autocuidado ^3^.

As representações sociais são entendidas como um conjunto de explicações, crenças e ideias que possibilitam caracterizar ou identificar um dado acontecimento, pessoa ou objeto, e, por serem resultantes da interação social, são formas de conhecimentos construídas e compartilhadas por um grupo social, responsáveis pela comunicação entre as pessoas que o integram, informam comportamentos, valores, imagens e auxiliam na sua tomada de posição perante o objeto representado ^9^.

É no corpo e por meio do corpo que a dimensão simbólica das masculinidades se materializa e colabora com a busca pela (re)produção de corporalidades que contribuam para a concretização dos ideais impostos pelo modelo de masculinidade hegemônica, que dificultam o reconhecimento de necessidades de atenção à saúde, tornando-os mais propensos a condutas que podem interferir em suas condições de saúde e levá-los ao adoecimento ^10^. Nesse caso, a hegemonia alude a um ideal normativo e honrado de ser homem ^6^, com características de dominância, força e virilidade ^11^ que sucumbem quando o homem é acometido por um câncer de próstata.

As representações sociais de homens com câncer de próstata sobre aspectos do adoecimento permitem, potencialmente, explorar o que eles sabem e como sabem, além de quais efeitos advêm desse saber, exercício necessário à luz da epistemologia das representações sociais ^12^, o que incita o seguinte questionamento: quais são as representações sociais das causas do adoecimento por homens diagnosticados com câncer de próstata?

Explorar tais vertentes contribui para que sejam propostas estratégias de cuidado focadas em uma assistência integral, além de possibilitar a discussão de como tais representações repercutem na prática do autocuidado ^13^.

O objetivo deste estudo é compreender as causas do adoecimento de câncer de próstata nas representações de homens acometidos por esse tipo de câncer e suas repercussões no autocuidado.

## Método

Estudo descritivo, qualitativo, baseado na Teoria das Representações Sociais ^9^. Seguiu-se as orientações metodológicas do instrumento *Consolidated Criteria for Reporting Qualitative Research* (COREQ) ^14^.

O campo foi o ambulatório de urologia da Unidade de Alta Complexidade em Oncologia (UNACON) do Hospital Universitário João de Barros Barreto (HUJBB), referência estadual para o controle do câncer no Estado do Pará.

Participaram 31 homens (27%) de um total de 118 com câncer de próstata, matriculados no HUJBB e atendidos no referido ambulatório. Interromperam-se as entrevistas quando os dados foram considerados empiricamente suficientes para atender aos objetivos da pesquisa ^15^ ^,^ ^16^.

Foram critérios de inclusão, ser maior de 18 anos com diagnóstico de câncer de próstata, com, no mínimo, seis meses de tratamento ambulatorial, domiciliados na Região Norte. O tempo mínimo se justifica pela convivência do participante com as rotinas da instituição, o diagnóstico da doença e a terapia instituída.

Adotou-se como critérios de exclusão, pessoas com alterações cognitivas ou dificuldades de comunicação que impedissem a participação nas entrevistas e quem não estivesse presente no campo no período da coleta de dados. Não houve exclusões.

A pesquisa foi desenvolvida por profissionais graduados e pós-graduados em enfermagem, sendo três mulheres e um homem, sendo o responsável pela coleta de dados. Esse pesquisador tinha contato prévio com o campo e experiência na técnica de coleta aplicada. A coleta dos dados ocorreu de abril a junho de 2022, por entrevistas individuais semiestruturadas, com roteiro composto por perguntas fechadas para identificação do perfil sociodemográfico, e seis perguntas abertas para a apreensão do objeto de estudo, que versaram sobre o que eles sabem sobre a doença, como se sentiu quando recebeu o diagnóstico, por que acha que teve a doença, como é conviver com câncer de próstata, como se cuida, do que precisa para se autocuidar. A duração média foi de 30 a 40 minutos. As entrevistas ocorreram nas terças-feiras pela manhã, nos dias de atendimento ambulatorial de urologia.

A abordagem dos participantes foi por conveniência, nos dias das consultas ambulatoriais, não interferindo nas rotinas da unidade e dos participantes. Ao final da consulta com o urologista fez-se os convites mediante apresentação do objetivo da pesquisa. Os que concordaram, foram direcionados a uma sala, com garantia de privacidade, explicando-se a pesquisa, os objetivos, benefícios e riscos, e os mecanismos para minimizá-los. Solicitou-se permissão para gravação das entrevistas em aparelho eletrônico, iniciadas após a assinatura do Termo de Consentimento Livre e Esclarecido (TCLE).

Os dados do perfil foram tratados com recursos da estatística simples e percentual. As entrevistas foram transcritas na íntegra, segundo os critérios para elaboração do *corpus* e análise lexicográfica do ALCESTE, versão 2012 (https://www.image-zafar.com/Logiciel.html), para posterior análise e interpretação à luz da Teoria das Representações Sociais.

O software auxilia na análise de conteúdos textuais densos com sentidos diversos, obtidos a partir de textos discursivos, denominados de Unidade de Contexto Inicial (UCI), cada qual correspondendo a cada entrevista. Organiza e sintetiza as informações com base metodológica e abordagem conceitual dos mundos lexicais. Os dados são organizados por análises estatísticas e matemáticas, fornecendo o número de classes, suas afinidades e diferenças, formas radicais e palavras associadas com seus valores de *phi* ^17^. Essa análise gera figuras chamadas de dendrograma, que mostram as classes lexicais.

Nos dendrogramas se organizam as palavras de acordo com os radicais que expressam os mundos lexicais dos participantes e suas respectivas associações estatísticas, cujos conteúdos constam nas Unidades de Contexto Elementar (UCE), que são os excertos dos discursos que lhes servem de explicação. Neste artigo, será tratada a classe lexical 2, que aborda o objeto do estudo em tela, que integra o dendrograma da Classificação Hierárquica Descendente (CHD).

A pesquisa foi aprovada pelo Comitê de Ética em Pesquisa da Universidade do Estado do Pará em 27 de janeiro de 2022, obedecendo-se à *Resolução nº 466/2012* do Conselho Nacional de Saúde. Os participantes foram identificados por códigos alfanuméricos, seguindo-se a normatização do software usado na análise.

## Resultados

Dos 31 homens que participaram do estudo, 11 estão na faixa etária de 65 a 69 anos (frequência relativa: 35,6%); 23 são casados (frequência relativa: 74,2%); 18 procedentes do interior do estado (frequência relativa: 58,1%); 18 apresentavam Ensino Fundamental incompleto (frequência relativa: 56,3%); 18 eram aposentados (frequência relativa: 58,1%); 18 possuíam renda familiar de um a dois salários mínimos (frequência relativa: 58,1%) e 17 eram evangélicos (frequência relativa: 54,8%).

O ALCESTE dividiu os textos das 31 UCI em 791 UCE, com aproveitamento de 75% do material analisado. As causas do adoecimento de câncer de próstata foram evidenciadas na classe lexical 2, que se organizou com 83 palavras analisáveis, 147 UCE e 18% de retenção de conteúdo do *corpus* analisado. Considerando as palavras de maior associação estatística (*phi*), conforme a CHD (Tabela 1), identifica-se que o léxico de maior *phi* foi representado pelas palavras “pegar”, seguido por “achar”, “fumar”, “homem”, entre outras que apontam ser possíveis causas relacionadas aos hábitos de vida que levaram ao desenvolvimento do câncer de próstata. Conforme indicação do software, as UCE mais significativas dessa classe emergiram das UCI 30 e 26, cujos participantes têm renda familiar de até dois salários mínimos e Ensino Fundamental incompleto.

**Tabela 1:** Classificação hierárquica descendente (CDH) da classe 2, oriunda do ALCESTE. Belém, Pará, Brasil (N = 31).

Palavra	*Phi*
Pegar	0,42
Acho	0,34
Fumei	0,31
Homem	0,27
Doença	0,24
Liga	0,22
Saúde	0,22
Beber	0,21
Motivo	0,21
Peguei	0,20
Medico	0,20
Vai	0,20
Errada	0,18
Pode	0,17
Homens	0,17
Mulheres	0,16
Metia	0,16
Cigarro	0,16
Exames	0,15
Tivesse	0,14

As UCE indicam as relações que os participantes estabelecem entre comportamentos de autocuidado e a possibilidade de adoecer de câncer de próstata.

“*Eu acho que não é todo homem que pode ter o câncer de próstata, porque se ele crescer fazendo o que é certo, buscando sempre remédio, médico, não fumar e não beber, ele não vai pegar, depende muito da maneira como ele vive*” (UCI nº 5).

“*Eu acho que todo homem pode ter o câncer de próstata, mas só vai ter aquele que não se cuida, que não liga para a saúde, que nem eu. Quando a gente é jovem, achamos que nada vai acontecer, quando nos atentamos, a doença já está em nós*” (UCI nº 20).

“*Para descobrir logo, a gente tem que ficar atento com nossa saúde, digo isso porque estou com câncer, mas antes, eu nem ligava para nada, nós, homens, achamos que nunca vai acontecer isso com a gente, ficamos a maior parte do tempo preocupados com família, casa, trabalho e esquecemos de nós mesmo*” (UCI nº 12).

O alcoolismo e o tabagismo são práticas mencionadas pelos homens como principais causas do câncer de próstata.

“*Eu fumei muito durante minha vida, e sempre quando olhava na carteira de cigarro, lá dizia que a gente pode pegar câncer, tinha umas fotos bem feias inclusive, só que a gente acha que nunca vai acontecer com a gente*” (UCI nº 31).

“*Eu não sei explicar direito porque tive essa doença, na verdade, é difícil explicar como essa doença apareceu em mim, mas sempre quando eu olhava na carteira de cigarro que eu fumava, lá dizia que o cigarro pode dar câncer*” (UCI nº 5).

“*Eu não sei falar porque eu tive isso* [câncer de próstata]*, eu não acho que todo homem pode ter esse câncer, porque eu tenho vários amigos que não têm, mas eu acho que aquelas pessoas que levam uma vida mais errada: bebem, fumam, podem ter mais chance de ter o câncer de próstata*” (UCI nº 13).

Algumas UCE indicam que o desenvolvimento do câncer de próstata está relacionado com um tipo de vida desregrada que os participantes tinham antes do diagnóstico, imputando a promiscuidade sexual como causa da doença.

“*Acho que peguei essa doença devido ao tipo de vida que eu levava no passado, quando eu era mais jovem, me envolvia com muitas mulheres, era igual um cachorro, saia de uma e me envolvia com a outra*” (UCI nº 19).

“*Quando mais jovem, eu vivia fumando e bebendo muito, passei mais de trinta anos da minha vida fazendo isso, não ligava muito para essas coisas de saúde, o que eu gostava mesmo era de mulher e farra*” (UCI nº 11).

“*A maioria dos homens tem possibilidade de pegar o câncer de próstata, uns devido pegarem doenças de outras mulheres, por não usarem preservativo, e outros pegam por não cuidarem da saúde, ou quando alguém da família tem o câncer de próstata*” (UCI nº 19).

Evidenciam-se também associações do câncer de próstata a um histórico familiar e à cor da pele.

“*Além disso, tem a questão do meu pai, acho que o fato de ele ter câncer ajudou a aparecer em mim também, porque a maioria dos meus amigos comentaram que alguém da família deles tinha pegado isso ou outro tipo de câncer*” (UCI nº 22).

“*Mas como eu sou homem, seja alguém para me derrubar ou uma doença, eu estou esperando como Satanás espera Deus. Eu acho que todo homem pode ter câncer de próstata e a maioria dos homens têm, principalmente a gente que é moreno, eu vi numa reportagem que o moreno é mais fácil de pegar a doença*” (UCI nº 30).

## Discussão

Os léxicos da CHD e as UCE que lhes servem de explicação conduzem a uma reflexão contextual, com elementos morais, éticos e sociopolíticos que envolvem a doença, quando os participantes aludem a opções classificadas por eles como erradas, na condução de seus comportamentos de autocuidado. Comportar-se de forma errada é uma classificação que decorre de uma avaliação moral, de certo e errado, mas também ética, de fazer bem ou mal ^18^, no caso, a si mesmo. O dualismo entre o certo e o errado se evidencia nos resultados como se houvesse um caminho certo a ser trilhado, mas que por opções erradas, adoeceram, o que mostra uma forte carga moral associada às explicações sobre por que adoeceram de câncer de próstata.

Essa carga moral que atribui à vítima a responsabilidade pelo seu adoecimento em razão das suas escolhas gera culpa e mostra-se injusta, pois promove um apagamento dos determinantes sociais da saúde que incidem nos fatores que levam ao adoecimento, pois as decisões sobre si e sua saúde estão relacionadas à posição sociocultural e educacional e ao acesso a bens e serviços dos cidadãos; logo, esse tipo de moralidade precisa ser bem evidenciado para que a punição às vítimas não seja alimentada por discursos de descuidos pessoais ^19^. Sobre o autocuidado masculino, existe um histórico de distanciamento dos serviços de saúde, o que dificulta os cuidados preventivos ^20^, sendo comum emergir a autoatribuição de culpa pela negligência ao autocuidado.

Evidenciam-se também nos resultados categorias morais afeitas ao discurso cristão, de tomar decisões corretas para seguir um caminho de vida orientado por condutas normativas, como também reflexões sobre as ações e suas consequências para si, com interpretações que afloram crenças e estereótipos. No cristianismo, o elo entre pecado e punição explica a doença como um castigo divino. Os resultados desta pesquisa mostram que as explicações sobre as causas do câncer de próstata veiculam representações edificadas em uma ideologia conservadora que culpabiliza quem não segue os padrões dominantes ^21^. A doença como expiação dos pecados, cuja origem está em comportamentos desregrados e não aprovados socialmente, carrega um julgamento moral relacionado ao comportamento de seus acometidos ^22^.

Acessar essas moralidades que atravessam as causas do adoecimento de câncer de próstata reforça que o cuidado com o corpo é uma ação complexa, que não se reduz às dimensões biológicas, mas, por significados sociais, se tornando um elemento histórico, político e cultural ^23^ ^,^ ^24^.

Historicamente, o homem reprime suas necessidades de cuidados, levado pelo imaginário social e pelas vinculações culturais e históricas do que é ser homem, traduzidas em sentimento de invulnerabilidade, resistência e impossibilidade de adoecimento, afastando-o das práticas de cuidados e prevenção de doenças ^25^.

A masculinidade ainda é fortemente ligada à cultura machista que dita as regras do comportamento masculino, indicando que ele é capaz de fazer tudo sem sofrer danos, enquadrando-o no estereótipo de um ser forte, que não carece de cuidados ^26^, buscando ajuda somente na presença de sintomas agudos, com uma visão curativa em detrimento da preventiva.

Pesquisa de representações sociais realizada com estudantes de medicina e enfermagem indica que ainda permanecem ideias de que a mulher está mais atenta aos cuidados com a saúde em termos de prevenção, e os homens cuidam da saúde mediante a doença ^27^. De igual modo, uma pesquisa com 29 homens pós-tratamento de câncer de próstata no sudeste da Inglaterra resultou em uma análise sobre as relações estabelecidas entre a saúde do homem e a masculinidade hegemônica ^28^, e uma metassíntese qualitativa evidencia que a defesa da masculinidade hegemônica gera implicações para o cuidado de saúde aos homens, com graves consequências, e reverbera na negligência de cuidados à saúde ^29^. Esses resultados vêm ao encontro do achado da pesquisa em tela neste artigo, de que a forma como os homens (não) se autocuidam segue concepções arcaicas sobre o que é ser homem, fortemente ancorada em uma ideologia machista e patriarcal, presente e difundida na sociedade, com reflexos práticos na vida cotidiana.

O medo de procurar os serviços de saúde e descobrirem-se doentes dificultou os participantes de se engajarem nas práticas de controle de cuidados em saúde, visto que o diagnóstico de doenças crônicas pode culminar em afastamento dos trabalhos, impactar na renda familiar e comprometer a imagem retrógada do homem como único provedor do lar, assemelhando-se ao estudo que mostrou como o tratamento de câncer de próstata é entendido como perda de status moral e interrupção da masculinidade, por homens do sudeste da Inglaterra ^28^.

O trabalho e a geração de renda desempenham um importante papel na constituição da identidade masculina, sua perda representa uma desvalorização social do “homem de verdade” ^10^. O trabalho é uma preocupação central pela questão da sobrevivência em uma sociedade capitalista, principalmente daqueles com baixa renda, como os homens deste estudo, cujo sustento da casa e da família se sobrepõe ao seu autocuidado.

A construção do universo masculino no imaginário social fornece uma base cultural que permeia a vida em sociedade e estabelece valores e crenças que servem como parâmetros a serem seguidos ^30^ ^,^ ^31^. Uma das funções das representações sociais é a de orientação, que direciona os comportamentos dos sujeitos de acordo com a representação que ele constrói sobre determinado objeto ^32^. Logo, se a representação social da masculinidade gera uma imagem de força para a figura do homem, tanto do ponto de vista psíquico-emocional quanto físico, por exemplo, a ele cabe abster-se de cuidados para consigo, à luz da imagem criada por uma sociedade impregnada por dogmas desse modelo de masculinidade.

Culpa e fracasso na experiência do adoecimento são palavras que envolvem uma dimensão de desvio pessoal, cujo resultado é a doença, e evocam valores morais distintos. À luz do discurso cristão, a culpa pode relacionar o surgimento da doença como castigo pela pessoa não seguir determinados padrões de cuidados. O fracasso, por sua vez, é o resultado de uma frustração de expectativa quanto à capacidade de evitar ou sobreviver à doença ^19^ ^,^ ^33^.

A palavra culpa vem do latim culpa, que significa a ocorrência de um erro, desacordo, engano. Os muitos paradigmas que permeiam o diagnóstico do câncer, sua possível gravidade e consequências, desencadeiam sentimento de culpa, visto o paciente acreditar que fez algo errado durante a vida, seja em ações, palavras ou práticas que culminam na doença ^34^. Ao considerar que fez algo errado ou deixou de fazer o que deveria ser feito, a pessoa se sente responsável pelo o que está passando, como uma forma de moldar sua vida de acordo com a lei do merecimento ^35^ ^,^ ^36^.

Ao buscar desmistificar certas doenças, como a tuberculose, o câncer e a aids, Sontag ^33^ afirma que, quanto menos conhecidos são os mecanismos das doenças em sua totalidade, mais o imaginário toma conta de explicá-las e, quando o faz, sempre surgem respostas de cunho moral, religioso ou, até mesmo, ideológico, que objetivam entender e justificar o ocorrido, ou seja, buscar uma causa para aquela consequência, neste caso, o câncer de próstata. Assim, as fotos e informações contidas nas carteiras de cigarros serviram de objetivações e ancoragens do câncer como doença que mata e que pode ser consequência de tais hábitos - dimensão da informação e da imagem, categorias importantes na formação de representações sociais ^9^.

Embora seja uma doença antiga e há muito tempo estudada no campo biomédico, ainda hoje há um intenso debate acerca dos fatores que compõem a base etiológica das neoplasias. Cada vez mais, constata-se a complexidade dessa enfermidade, marcada por causas multivariadas e multideterminadas, que incluem fatores internos, relacionados à hereditariedade, e externos ao organismo, como o tabagismo, alimentação inadequada, ausência de atividades físicas e à radiação ^2^.

Com relação ao desenvolvimento do câncer de próstata, algumas UCE remetem à ideia de que a doença possa ser transmitida, evidenciada pelo léxico “peguei”. Esse fato remonta às ideias elaboradas e disseminadas entre o século XIX e início do século XX, no qual o câncer era considerado contagioso e associado à falta de limpeza, à sujeira física e ao comportamento moral degradante, atribuindo aos doentes a culpa pelo próprio adoecimento ^37^.

Diante do incômodo produzido pelo desconhecimento de propriedades detalhadas dos cânceres, pesquisadores recorriam a modelos de compreensão de outras doenças com elevada prevalência e incidência na época, em especial, tuberculose, sífilis e hanseníase. As tentativas de aplicação desses modelos ao estudo dos cânceres contribuíram para o desenvolvimento de práticas segregacionistas como, por exemplo, o isolamento social das pessoas com câncer, a internação compulsória e a criação de asilos e, consequentemente, a estigmatização dos enfermos na sociedade ^38^.

Um dos sentimentos citados ante o diagnóstico do câncer é o medo, que pode se manifestar devido à relação da doença com morte e ao desconhecimento de sua etiologia, haja vista que não se deve desconsiderar reminiscências de ideias que relacionam o câncer à transmissibilidade ^33^. A ideia da transmissibilidade pode ser apreendida na UCE da UCI ^19^.

A vida pregressa de promiscuidade sexual, traduzida como a de um cachorro, anuncia a possibilidade de uma objetivação e ancoragem sobre uma tipologia comportamental do homem, aproximada com a de um animal irracional, visto que ao atingir sua maturidade sexual, o cachorro macho vive um cio constante quando percebe a presença de fêmeas férteis em seu ambiente, ou seja, sua natureza o leva ao encontro das fêmeas para garantir a sobrevivência da espécie.

O homem, por se envolver com muitas mulheres, se traduz como um cachorro, como se essa busca fosse natural do homem. Observa-se, no entanto, que esse comportamento do homem é cultural e com forte relação com a formação social da masculinidade. Vê-se, aí, mais uma vez, a força do discurso patriarcal incidindo sobre as representações da masculinidade.

Outra vertente importante, ainda sobre esse resultado, é que na interpretação sobre a origem da doença identifica-se uma relação de envolvimento com variadas mulheres, o que leva ao pressuposto de que uma das causas do câncer de próstata possa estar relacionada à diversidade de mulheres no relacionamento e não necessariamente ao comportamento masculino visto como natural. Essa relação deve ser mais bem explorada em pesquisas futuras, já que não há dados suficientes para aprofundar essa vertente nos resultados desta pesquisa.

As representações sociais são fenômenos específicos que estão relacionados com um modo particular de compreender e de se comunicar, formando o conjunto de elementos que fazem parte do senso comum ^39^. Com base nesse entendimento, pode-se destacar que os meios de comunicação, especialmente os de massa, atuam como elemento cultural importante na formação do senso comum, em que o conteúdo veiculado na mídia contribui para criar uma realidade que auxilia na compreensão de elementos do cotidiano, nesse caso, difundindo imagens, crenças, informações e opiniões. Identifica-se na citação de um dos participantes, por exemplo, a cor da pele relacionada ao câncer de próstata difundida em uma reportagem (UCI nº 30).

Relacionada ao câncer de próstata, a relação entre a cor da pele e a prevalência é descrita na literatura como sendo mais comum em homens da população negra do que em brancos ^40^ ^,^ ^41^. Esses dados mostram que as informações reificadas que se disseminam por meio dos meios de comunicação foram incorporadas ao senso comum de homens com câncer de próstata, contribuindo assim para a construção de novos saberes sobre a doença que estavam vivenciando.

A mídia tem um papel fundamental para o conhecimento do senso comum, pois, ao popularizar para o leigo os conhecimentos produzidos pela ciência, age na produção e veiculação de representações sociais assim como transmite códigos normativos de comunicação e conduta ^9^. Ainda mais, as representações sociais buscam explicitar como os saberes, em nível social, permitem à coletividade processar um dado conhecimento veiculado pela mídia, transformando-o em uma propriedade impessoal, pública, que permite a cada indivíduo o manuseio e a utilização de forma coerente com os valores e as motivações sociais da coletividade a qual pertence ^39^.

Um ponto comum nos resultados exemplificados em algumas UCE diz respeito aos homens atribuírem o desenvolvimento do câncer de próstata ao fato de alguém da família já ter tido diagnóstico, o que contribui para a elaboração e manutenção da noção de câncer como uma doença familiar.

A suscetibilidade hereditária é um dos fatores de risco para o desenvolvimento do câncer de próstata, que aumenta 2,2 vezes quando um parente de 1º grau é acometido pelo câncer de próstata, 4,9 vezes quando dois parentes de 1º grau têm câncer e 10,9 vezes quando três parentes de 1º grau têm a doença ^2^ ^,^ ^42^ ^,^ ^43^. Além da genética, a religiosidade também serve de grade de leitura para explicar o câncer de próstata, como um desígnio de algo/alguém superior, divino, com um objetivo específico em sua vida. Isso mostra que o câncer ainda é carregado de fortes estigmas, significados e símbolos negativos no meio social, por esse motivo, muitos tentam amenizar ou enfrentar essa situação com explicações místicas para a doença ^3^. Estudos realizados com pacientes oncológicos em cuidados paliativos mostram a predominância de explicações de cunhos espirituais, com ênfase na vontade de Deus como aspecto decisivo do adoecimento, estratégias adotadas para o enfrentamento do câncer, contribuindo para a construção de uma estrutura que permite refletir sobre os significados e sentidos da existência da doença, fornecendo base para a compreensão da nova realidade que estavam vivenciando ^3^ ^,^ ^44^ ^,^ ^45^.

As representações sociais contribuem na normalização dos objetos, comportamentos, acontecimentos, crenças e valores que circundam a vivência de cada sujeito, e que lhe permite explicar ou dar sentido a um fenômeno desconhecido, não familiar, em um só modelo de significação, para que assim se torne compreensível em seu meio social ^39^ ^,^ ^46^. Dessa forma, os aspectos religiosos para os homens com câncer de próstata assumiram a função de tornar familiar uma realidade desconhecida, possibilitando uma explicação do motivo que os levou ao quadro oncológico que estão enfrentando.

As representações das causas que levaram ao adoecimento por câncer de próstata mostram influências de valores judaico-cristãos e do patriarcado. A grade de leitura punitiva por uma vida vivida antes do adoecimento ^47^ contribui para reforçar a culpa dos homens por se sentirem homens e serem homens à imagem do que se espera numa sociedade capitalista e patriarcal.

A compreensão das causas atribuídas ao adoecimento sob a ótica das representações sociais de homens que a vivenciam contribui para reflexões que possibilitem elucidar ainda mais as influências de elementos psicossocioculturais que incidem no pensamento e na ação de pessoas, em face aos fenômenos que envolvem a saúde, a doença e o cuidado, evidenciando a forte relação entre esses elementos e suas implicações sociais.

Tais reflexões importam para o desenvolvimento profissional na atenção oncológica, mais precisamente dos enfermeiros, visto que são os primeiros profissionais a estabelecer contato com o paciente oncológico na rede de atenção, de modo que ao prestar a assistência seja considerado o conhecimento prévio sobre a doença, o que permite entender as necessidades percebidas e expressas por meio dos anseios, temores e conflitos ante o diagnóstico do câncer de próstata, para que assim, sejam feitos atendimentos humanizados e se estabeleçam medidas de cuidados preventivas mais eficazes e efetivas, com estímulos ao autocuidado, entendendo que as implicações de cuidar da saúde do homem transcendem à dimensão biológica, mas sim, alcança a dimensão político-social do corpo.

Assume-se como limitação do estudo, ter sido realizado em apenas um dos dois centros de referências oncológicas públicas do Estado, não permitindo ampliação de participantes com mais variáveis de perfil. Entretanto, com base nos resultados apresentados, novos estudos em outros cenários podem contribuir para ampliar a reflexão e compreensão das representações sociais dos homens com câncer de próstata.

## Considerações finais

A ingestão de bebida alcoólica, o tabagismo, o não autocuidado e a promiscuidade sexual foram os principais comportamentos entendidos como causas do câncer de próstata. Os homens se atribuem a responsabilidade e se culpam pela doença, em razão de comportamentos que a moral social dita como certos. Os pensamentos judaico-cristão e patriarcal fundamentam essa culpa e evidenciam que ainda circulam representações sociais de que desenvolver doenças crônicas é uma punição pelas opções pessoais não normativas, o que reforça pensamentos de senso comum sobre a culpa do doente pelo adoecimento. Não seguir condutas moralmente corretas e não se afinar à ideia de homem forte os desviam do que seriam ou deveriam ser práticas de autocuidado preventivas. O discurso religioso diminui a carga sociopolítica das vulnerabilidades masculinas, quando a interpretação das causas do câncer de próstata se organiza em representações sociais que o justificam e ainda reafirmam e se ancoram nos sustentáculos que mantêm a sociedade capitalista e patriarcal.
